# Incidental Detection of a Right-to-Left Atrial Shunt Without Pulmonary Hypertension

**DOI:** 10.7759/cureus.115168

**Published:** 2026-08-25

**Authors:** Shiv Malhotra, Calista Kee, Leeza Peerzada, William M Strub

**Affiliations:** 1 Radiology, CUNY School of Medicine, New York, USA; 2 Radiology, University of Cincinnati College of Medicine, Cincinnati, USA

**Keywords:** dyspnea, hemodynamics, incidental finding, intracardiac shunt, right-to-left shunting

## Abstract

Right-to-left interatrial shunting permits venous blood to bypass the pulmonary circulation and enter the systemic circulation, potentially contributing to hypoxemia and paradoxical embolization. Although sustained right-to-left shunting is commonly associated with elevated right-sided or pulmonary arterial pressures, it may also occur in patients with normal pulmonary pressures because of transient pressure gradients or preferential flow across an interatrial communication. We present a 57-year-old man without known cardiac or pulmonary disease who developed progressive exertional dyspnea. Because his D-dimer result did not exclude pulmonary embolism, planar ventilation/perfusion imaging was performed following intravenous administration of technetium-99m macroaggregated albumin. Pulmonary perfusion was normal without any segmental defects; however, unexpected radiotracer uptake was identified in the brain and kidneys. This raised suspicion for a right-to-left shunt. CT pulmonary angiography subsequently demonstrated contrast passage from the right atrium to the left atrium. Transthoracic echocardiography with a bubble study confirmed a large right-to-left shunt at the atrial level, with normal right ventricular size and thickness, an estimated systolic pulmonary artery pressure of 15 mmHg, and an estimated right atrial pressure of 8 mmHg. The patient was referred for cardiology follow-up. This case demonstrates that systemic uptake of technetium-99m macroaggregated albumin during lung perfusion imaging should prompt evaluation for right-to-left shunting, even in the absence of pulmonary hypertension.

## Introduction

Right-to-left shunting results from an abnormal pathway that permits deoxygenated blood from the venous system to bypass the lungs and directly enter systemic circulation. Cardiac right-to-left shunts across the interatrial septum are typically associated with congenital heart defects and elevated pulmonary arterial pressures, thereby driving hemodynamic flow [1]. The case presented is a unique scenario in which a right-to-left atrial shunt was discovered incidentally in a patient with suspected pulmonary embolism, in the absence of pulmonary hypertension, after Technetium-99m (Tc-99m) uptake was visualized in the brain and kidneys. Right-to-left shunting without associated pulmonary hypertension is rare in clinical scenarios, with several potential causes, such as abnormal pressure gradients or anatomical variations, having been reported in the literature [2-5]. Recognition is clinically important because right-to-left interatrial shunting may contribute to hypoxemia and provide a pathway for paradoxical systemic embolization, prompting targeted cardiac evaluation. This case highlights the importance of recognizing incidental findings and considering a wide gamut of atypical physiological processes that could explain unexpected discoveries during routine imaging.

## Case presentation

**Figure 1:**
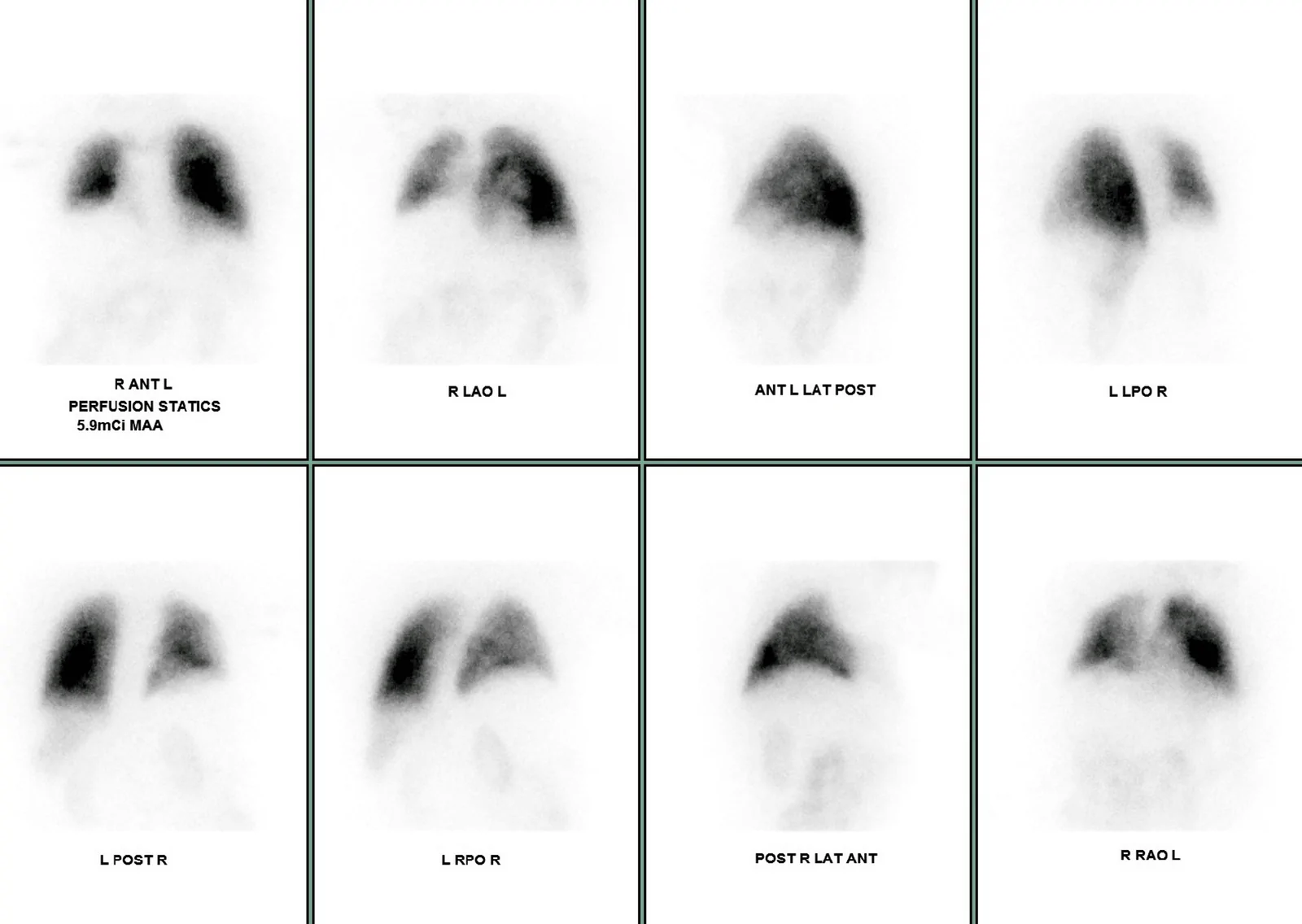
Ventilation/Perfusion (V/Q) scan of the patient demonstrating adequate uptake of Tc-99m macroaggregated albumin (MAA) radiotracer in the lungs without any segmental perfusion defects

A 57-year-old man without any prior history of cardiac or pulmonary disease presented to the clinic with progressive shortness of breath upon exertion. Further physical examination was unremarkable, with clear lungs, normal heart sounds, and warm, dry, and acyanotic skin with normal turgor. There was no other notable surgical or smoking history noted for the patient. Upon suspicion of a possible pulmonary embolism, a D-dimer test was performed and did not exclude pulmonary embolism, prompting follow-up radiological examination. Additional pretest-probability variables and the precise duration of symptoms were not available in the source documentation. Based on the ordering physician's preference, a ventilation/perfusion (V/Q) scan and chest radiograph were the initial tests performed, with 5.9 millicuries of technetium-99m macroaggregated albumin (Tc-99m MAA) radiotracer injected intravenously prior to planar imaging. Upon review of imaging, lung perfusion appeared to be normal with no segmental defects noted, ruling out a pulmonary embolism (Figure 1).

**Figure 2:**
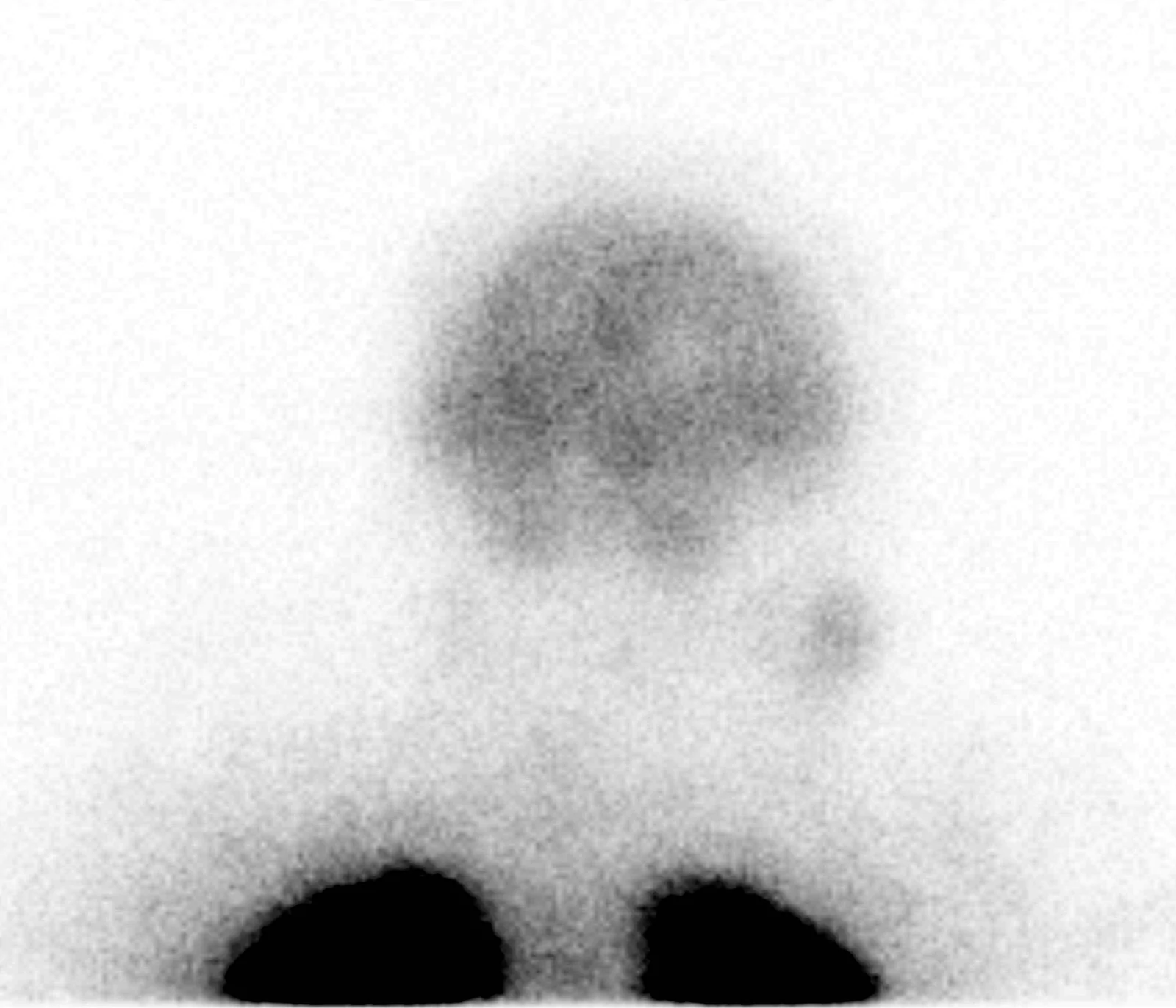
Planar perfusion scintigraphic image demonstrating uptake of Tc-99m macroaggregated albumin (MAA) radiotracer in the brain, raising suspicion for a right-to-left shunt

However, radiotracer uptake was incidentally noted in the brain (Figure 2) and kidneys, suggesting a possible right-to-left intracardiac shunt.

**Figure 3:**
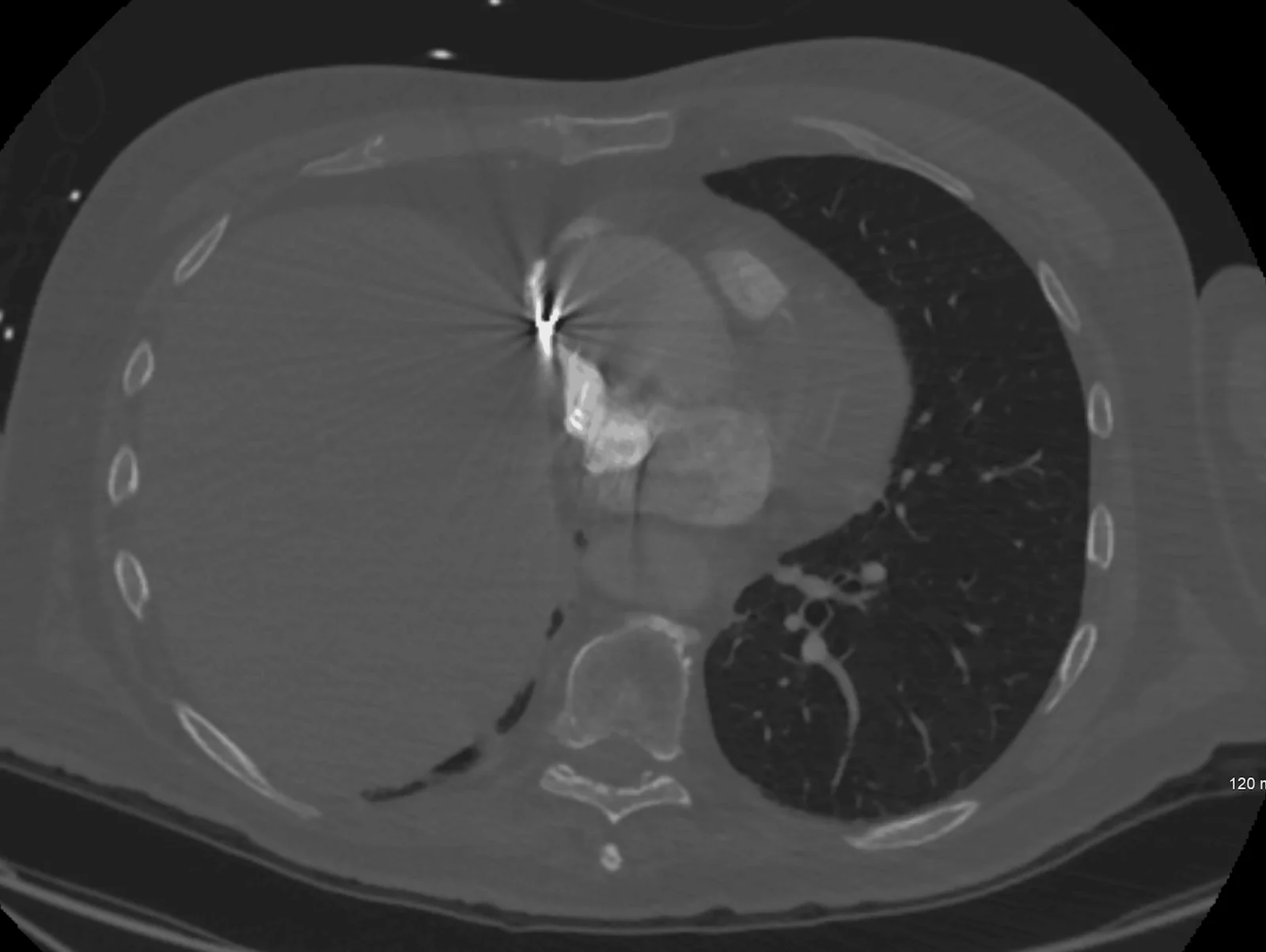
Contrast-enhanced CT pulmonary angiographic image at the level of the atria demonstrating a contrast jet from the right atrium to the left atrium, consistent with right-to-left interatrial shunting

A subsequently performed CT pulmonary angiogram (CTPA) confirmed the presence of a right-to-left atrial shunt, with a contrast jet seen directly flowing from the right atrium to the left atrium (Figure 3).

A limited transthoracic echocardiogram (TTE) for right ventricle size, systolic pulmonary artery pressure (SPAP), right atrial pressure (RAP), and intracardiac shunt detection with a bubble study was then performed. This confirmed the presence of a large right-to-left shunt at the atrial level, along with a right ventricle that appeared normal in size and thickness, a normal SPAP estimated to be 15 mmHg, and a RAP just within normal limits of 8 mmHg. The patient was sent for follow-up by cardiac care services for further management and follow-up.

## Discussion

A right-to-left shunt is an abnormal communication that permits deoxygenated blood to flow from the right to the left side of the heart, and ultimately to systemic circulation. There are a variety of different levels and sequelae at which such a shunt can arise intracardially, either directly or indirectly. Directly, Tetralogy of Fallot, pulmonary atresia, double-outlet right ventricle, complete transposition of the great vessels, and truncus arteriosus can all cause right-to-left shunting due to sequelae from congenital anatomical abnormalities. Indirectly, intracardiac left-to-right shunts, such as an atrial septal defect (ASD), can eventually reverse to right-to-left shunts due to a progressive increase in pressure in the right chambers of the heart or pulmonary arterial system. A patent foramen ovale (PFO) may also permit right-to-left shunting when right atrial pressure transiently or persistently exceeds left atrial pressure or when blood is preferentially directed across the communication. The most prominent example of shunt reversal is Eisenmenger’s syndrome, where an ASD initially causes a left-to-right shunt, and if left untreated, it causes pulmonary vascular injury and a gradual increase in pulmonary blood pressure, ultimately precipitating a reversal towards a right-to-left shunt [1]. While right-to-left shunts in adults are typically seen as a result of elevated pulmonary arterial pressures driving the reversal of a left-to-right shunt, this patient was found to have right-to-left shunting at the atrial level, but without an elevated SPAP or right atrial pressure. This highlights the uniqueness of the possible hemodynamics at play with our patient, as the driving force for blood from the right to the left atrium is unknown.

There have been two major theories published in the literature that explain potential causes of right-to-left direction of shunting without pulmonary hypertension. One theory is the presence of a systolic pressure gradient between the right and the left atrium. Pathologically, myxomas or metastatic disease of the right atrium, right ventricular infarction, tricuspid or pulmonic valve disease, and pericardial tamponade, among others, can all cause such a pressure gradient. Transiently, changes in posture or inspiration, coughing, or the Valsalva maneuver can all cause an elevated right atrial pressure in the absence of pulmonary hypertension.

In the absence of abnormal atrial pressure differences, another theory that describes right-to-left shunting is blood flow streaming preferentially from the inferior vena cava to an interatrial communication such as a PFO during systole. This can be caused by a persistent eustachian valve, which directs blood flow from the inferior vena cava across the atrial septum, and ultimately to systemic circulation in the embryo. In instances where the foramen ovale remains patent, a persistent eustachian valve can continue to direct blood flow from the inferior vena cava to systemic circulation as it did during embryogenesis, but with unoxygenated blood. Additionally, other anatomical or clinical abnormalities and surgical procedures can cause a displacement of the atrial septum to the horizontal axis, placing the interatrial communication directly in line with the stream of blood from the inferior vena cava into the right atrium. Among others, these include a dilatation of the ascending aorta, hemidiaphragmatic paralysis, pneumonectomy, and abdominal surgery [2-5].

Typically, when right-to-left atrial shunting is initially suspected, an echocardiogram with an agitated saline bubble study or contrast is performed. The echocardiogram is marked as positive for a right-to-left atrial shunt if contrast is seen in the left atrium during early ventricular systole. If positive, a follow-up transesophageal echocardiogram may be performed when confirmation or more detailed characterization of the interatrial communication in terms of size and shape is clinically required [6]. In the present case, the V/Q scan preceded echocardiography because it was obtained to evaluate suspected pulmonary embolism rather than a suspected intracardiac shunt. Right-to-left atrial shunts can also be incidentally detected, as seen with this patient, during lung perfusion imaging with Tc-99m MAA scintigraphy. Abnormal radiotracer uptake in organs such as the brain and kidneys suggests some blood flow circumventing pulmonary circulation and directly going into systemic circulation after intravenous injection of Tc-99m MAA [7].

Clinically speaking, right-to-left atrial shunting can result in hypoxemia due to the mixing of oxygenated and deoxygenated blood, leading to dyspnea, cyanosis, dizziness, and other consequences of low systemic arterial oxygen content [8,9]. The patient presented with dyspnea but was acyanotic on physical examination. There also are several other notable clinical risks of atrial right-to-left shunting clinicians should be aware of, including paradoxical embolisms and atrial arrhythmias [10]. Indications for closure depend on the type of interatrial communication and the clinical context. For ASDs, closure may be indicated for a significant net left-to-right shunt, commonly a pulmonary-to-systemic flow ratio (Qp) of at least 1.5:1 with right-sided enlargement, whereas PFO closure is generally considered under distinct clinical criteria such as selected cases of PFO-associated stroke [11,12].

## Conclusions

The case presented displays a unique clinical scenario of a patient with a suspected pulmonary embolism, upon which further workup displayed an incidental finding of a right-to-left atrial shunt on a V/Q scan after Tc-99m MAA uptake was noted in the brain and kidneys. Right-to-left atrial shunting was suggested by CTPA and confirmed with TTE, with a notable absence of pulmonary hypertension. The available studies did not definitively characterize the interatrial communication as a PFO or a specific type of ASD, and subsequent treatment and long-term outcome were unavailable. This made the exact mechanism of the shunt’s hemodynamics unclear, with several theories potentially being at play in the patient. Overall, this case underscores the importance of meticulous interpretation of imaging studies outside of the intended area, as incidental findings can lead to significant diagnostic discoveries. Furthermore, it highlights the need for further research on the pathophysiology and hemodynamics of right-to-left shunts.
